# Quantitative blush evaluator (QuBE) accurately quantifies microvascular dysfunction in patients with ST-Elevation Myocardial Infarction; comparison with Cardiovascular Magnetic Resonance

**DOI:** 10.1186/1532-429X-13-S1-O53

**Published:** 2011-02-02

**Authors:** Christian Hamilton-Craig, Italo Porto, Giovanni Luigi De Maria, Luigi Natale, Filippo Crea

**Affiliations:** 1 University of Queensland, Brisbane, Australia; 2 Catholic University of the Sacred Heart, Rome, Italy

## Introduction

### Background

After ST-elevation myocardial infarction (STEMI), microvascular obstruction (MVO) can be assessed using angiographic myocardial ‘blush’ grade (MBG), subject to interoperator variability. Quantitative blush evaluator (QuBE) is a free computer-calculated algorithm, with improved reproducibility. We aimed to compare QuBE in detecting MVO and its severity in comparison to the gold standard, cardiovascular magnetic resonance (CMR).

## Methods

Fifty-two STEMI treated with successful primary-PCI were enrolled. QuBE values were blindly calculated on post-procedure angiogram and patients divided in tertiles (ter) according to QuBE values. All patients underwent CMR (GE 1.5T) 4-7 days after STEMI, for quantitative assessment of infarct size (IS), area-at-risk (AAR), myocardial salvage index (MSI), MVO (both as first pass MVO (FP-MVO) and delayed-enhancement MVO (DE-MVO)), and presence of intramyocardial hemorrhage on T2-weighted sequences. Indices were measured blindly by two expert readers with SMCR level 3, using ReportCard (v4.0) software. LGE infarct size was quantified using threshold >5SD, T2 sequence >2SD as previously described. MVO was manually traced, and hemorrage identified as the dark band within bright signal on T2

## Results

**Figure 1:**
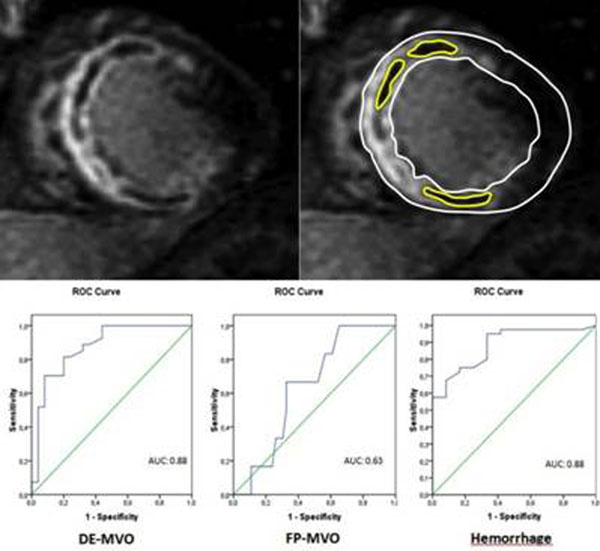
ROC curves for sensitivity and specificity of QuBE in detecting CMR derived values of DE-MVO, FP-MVO and hemorrhage

QuBE values were inversely related to IS, both as percentage (R: -0.4; p:0.001) and as mass (R:-0.4; p: 0.008), to DE-MVO, both as percentage (R: -0.7; p<0.001) and as mass (R: -0.7; p< 0.001), to FP-MVO (R:-0.4; p: 0.002), and positively related to MSI (R: 0.4; p: 0.007). Moreover patients with intramyocardial hemorrhage had significantly lower QuBE values (3.9; 3.5-8.0 vs 12.2; 8.2-16.0; p:0.001). At receiver operating characteristic (ROC) curve analysis, QuBE accounted for an area under the curve of 0.88 (CI 95% 0.7-0.9; p: 0.001) for both DE-MVO and haemorrhage detection. Figure 1.

## Conclusions

This is the first report to compare quantitative computer-based assessment of angiographic perfusion with comprehensive parameters of microvascular function, infarct size and salvage on early CMR in STEMI patients. We demonstrate the important finding that QuBE values correlate strongly with microvascular obstruction, Intramyocardial hemorrage and myocardial salvage by CMR. This validates the use of QuBE for assessment of tissue reperfusion in the clinical setting, and represents and important advancement in post-procedural evaluation of reperfusion in the cathlab. Our data suggest that a quantitative evaluation of myocardial blush after STEMI will accurately risk-stratify patients, and provide useful prognostic information, better informing decisions such as the selection of patients requiring further imaging (eg by CMR).

